# Streptavidin-Displaying *E. coli* Scaffolds Enable Target-Mediated Gold Nanoparticle Assembly for Colorimetric Biosensing

**DOI:** 10.3390/s26185965

**Published:** 2026-09-21

**Authors:** Po-Chih Wu, Shi-Yu Tseng, Shao-Yi Hou, Po-Shiuan Hsieh, Wen-Zhi Lin, Chin-Mao Hung

**Affiliations:** 1 Graduate Institute of Medical Sciences, College of Medicine, National Defense Medical University, Taipei City 114201, Taiwan; c862032000@gmail.com (P.-C.W.); pshsieh0465@gmail.com (P.-S.H.); 2 Graduate Institute of Microbiology and Immunology, College of Biomedical Sciences, National Defense Medical University, Taipei City 114201, Taiwan; 42120178james@gmail.com; 3 Department of Chemical Engineering and Biotechnology, National Taipei University of Technology, Taipei City 106344, Taiwan; f10955@ntut.edu.tw; 4 Institute of Preventive Medicine, National Defense Medical University, New Taipei City 237010, Taiwan

**Keywords:** point-of-care testing, bio-nanostructured hybrids, plasmonic coupling, multifunctional platform, pathogen detection, colorimetric biosensing

## Abstract

Enhancing the visual detection sensitivity of point-of-care (POC) optical sensors without resorting to complex, multistep chemical or enzymatic signal amplification remains a major challenge in sensor nanotechnology. Here, we present a multifunctional bio-nanostructured hybrid platform that integrates engineered *Escherichia coli* cellular scaffolds with antibody-functionalized gold nanoparticles (AuNPs) for high-sensitivity, solution-phase colorimetric sensing. By exploiting the biotin-streptavidin interaction through the Lpp-OmpA outer-membrane display system, the engineered bacterial cells serve as high-capacity, multivalent biological interfaces with a large effective surface area and high recognition-site density. Using SARS-CoV-2 nucleocapsid protein as a model biomarker, target-mediated bridging between AuNP-antibody probes and bacterial scaffolds promotes localized AuNP assembly and enhanced plasmonic coupling, resulting in a distinct visual color change within 30 min. The optimized assay achieved a naked-eye limit of detection (LOD) of 10^−12^ mol, representing a 10-fold sensitivity enhancement over conventional scaffold-free AuNP assays. This study provides a proof of concept for leveraging engineered living cells as multifunctional nanostructured templates. The modular, plug-and-play architecture of the biotin-streptavidin coupling interface highlights its potential for adaptation to clinical diagnostics and environmental monitoring.

## 1. Introduction

Point-of-care testing (POCT) has become crucial for improving access to timely diagnostic testing [1]. Unlike conventional laboratory-based methods, POCT enables diagnostic testing to be performed at or near the site of patient care, providing rapid results through a simplified workflow [2]. By reducing the time required to obtain analytical readouts, on-site rapid testing facilitates prompt decision-making, which is highly critical not only in clinical diagnostics but also in environmental safety monitoring, such as the on-site tracking of waterborne and foodborne pathogens in resource-limited or agricultural settings [3,4,5,6]. For both clinical and environmental applications, achieving rapid pathogen surveillance requires sensing platforms to meet the ASSURED criteria, which emphasize affordability, sensitivity, specificity, user-friendliness, rapidity and robustness, equipment-free operation, and deliverability to end-users [7]. These criteria have driven the development of portable diagnostic platforms designed to combine operational simplicity with adequate analytical sensitivity for broad-spectrum biosensing and environmental hazard detection.

Detection of severe acute respiratory syndrome coronavirus 2 (SARS-CoV-2) is a major focus in POCT development because SARS-CoV-2 is highly transmissible and often causes clinical manifestations that overlap with those of other respiratory viruses, including influenza viruses and respiratory syncytial virus (RSV) [8,9]. Therefore, the timely identification of SARS-CoV-2 infection is essential to clinical decision-making, patient isolation, and transmission control. In current clinical practice, real-time reverse transcription quantitative polymerase chain reaction (RT-qPCR) is considered the gold standard for SARS-CoV-2 detection because of its high sensitivity and specificity [10,11]. However, it requires specialized instrumentation, labor-intensive procedures, and trained personnel, which limits its application in point-of-care settings [12]. Compared with RT-qPCR, antigen-based detection methods provide a simpler testing workflow by reducing the need for specialized instrumentation. Among SARS-CoV-2 antigens, the nucleocapsid (N) protein is an attractive diagnostic target because its relatively conserved sequence may enable more consistent antigen recognition across circulating variants [13]. Accordingly, POCT strategies targeting the SARS-CoV-2 N protein represent a crucial approach to developing accessible and practical diagnostic platforms.

Colorimetric assays are well suited for POCT because they are simple to perform and enable rapid interpretation of test results through direct visual readout [14,15,16]. Lateral flow immunoassays have been widely developed for SARS-CoV-2 antigen detection. However, their nitrocellulose membrane-based format can introduce mass-transfer resistance, which may limit analyte transport and probe-target interactions and thereby restrict analytical sensitivity [17,18,19]. By contrast, solution-phase assay formats provide a homogeneous reaction environment that facilitates probe-target interactions, reducing transport limitations associated with porous membrane matrices. When combined with gold nanoparticles (AuNPs), target-induced changes in nanoparticle dispersion or aggregation can modulate localized surface plasmon resonance, converting molecular recognition events into visible signals that can be rapidly interpreted [20]. Despite these advantages, the analytical sensitivity of AuNP-based colorimetric assays remains limited, particularly at low target concentrations, where the resulting optical changes may be too subtle to reliably distinguish through visual inspection [21]. Although various signal amplification strategies, including chemical enhancement [22], enzyme-mediated amplification [23], and nanomaterial-assisted approaches [24], have been developed to improve colorimetric responses, many require additional reagents, complex modification procedures, or auxiliary functional materials. These additional requirements increase assay complexity and manufacturing costs, limiting the practical application of these approaches in POCT. Therefore, strategies that improve visual signal discrimination while preserving assay simplicity are still needed.

The incorporation of bacterial cells into sensing systems represents a promising strategy for enhancing analytical signals. The surface of *E. coli* can be modified through genetic engineering or chemical functionalization to display target-recognition molecules or to carry functional components, such as enzymes and nanomaterials, for signal generation and amplification. Because *E. coli* are micrometer-sized [25], their surfaces provide a relatively large biological interface that offers greater capacity for functional loading and improved accessibility. This increased surface capacity can enhance target capture, catalytic activity, and colorimetric signal transduction compared with freely dispersed probes or conventional planar supports [26,27,28]. In addition, the scalability and ease of *E. coli* culture support its use as a biological scaffold for assay development [26,29]. However, the application of *E. coli* scaffolds in assay development remains limited by the need for stable and adaptable surface functionalization. Weak or poorly controlled attachment of recognition elements or signal-generating components may reduce effective surface loading and compromise signal reproducibility, particularly in complex sample matrices [30,31]. Furthermore, when target-recognition elements must be redesigned and genetically fused for each new analyte, adaptation to new targets requires repeated construct generation and validation [32]. Therefore, a stable and reliable surface-coupling strategy is needed to achieve effective functionalization of *E. coli* scaffolds for sensitive colorimetric detection.

Biotin-streptavidin mediated coupling offers a promising approach for functionalizing *E. coli* surfaces. Streptavidin is a homotetrameric protein with four independent biotin-binding sites [33] and binds biotin through a strong noncovalent interaction, forming an effectively irreversible complex with a dissociation constant (K_d_) of approximately 10^−15^ M [34]. These binding properties have led to the widespread use of the biotin-streptavidin system for molecular immobilization and bioconjugation in analytical and detection platforms. In addition, the biotin-streptavidin system supports modular surface functionalization by coupling different biotinylated recognition antibodies through a common streptavidin interface [35]. In *E. coli*, outer membrane-anchoring systems such as Lpp-OmpA have been used to display functional streptavidin capable of binding biotinylated molecules [36], demonstrating the feasibility of using streptavidin-displaying *E. coli* as a functional scaffold. Importantly, this coupling strategy provides a modular recognition interface that allows target specificity to be readily reconfigured by simply exchanging the biotinylated recognition ligands, without requiring repeated genetic re-engineering or expression validation of the bacterial host. Such modularity could enable the same bacterial scaffold to be adapted to diverse sensing targets, ranging from environmental biohazard monitoring to clinical diagnostics. However, the application of such bacterial scaffolds in colorimetric biosensing remains relatively limited, and their potential to enhance colorimetric signals while maintaining assay simplicity has yet to be fully explored.

In the present study, we developed a solution-phase bio-nano colorimetric method for SARS-CoV-2 N protein detection by integrating streptavidin-displaying *E. coli* bacterial probes with antibody-functionalized AuNP probes. In this assay, the target antigen bridges the two probe types, promoting localized AuNP assembly on the bacterial scaffold and enhancing the resulting colorimetric signal. This study provides proof of concept that an engineered bacterial scaffold can organize AuNP-based probes and amplify colorimetric signals without relying on enzymatic reactions or complex signal-enhancement procedures. By utilizing the SARS-CoV-2 N protein as a rigorous biological model, this research establishes an adaptable, enzyme-free, and visually interpretable sensing archetype. Given its robust bioconjugation and modular architecture, this multifunctional bio-nano platform has potential for applications in clinical diagnostics and rapid, on-site environmental pathogen screening.

## 2. Materials and Methods

### 2.1. Materials and Buffers

The colloidal gold conjugation kit was purchased from Easyget Life Sciences (Taiwan). Bovine serum albumin (BSA; catalog no. A7906) was obtained from Sigma-Aldrich (St. Louis, MO, USA). Chloramphenicol (catalog no. CB0118) and L-arabinose (catalog no. AB0071L) were purchased from Bio Basic (Markham, ON, Canada). Isopropyl β-D-1-thiogalactopyranoside (IPTG, catalog no. I0355) was purchased from GenDEPOT (Barker, TX, USA). Sucrose (catalog no. 4072-01) and fluorescein biotin (catalog no. 80019) were obtained from J.T. Baker (Phillipsburg, NJ, USA) and Biotium (Fremont, CA, USA), respectively. The HF120 nitrocellulose membrane (catalog no. SHF1200225) was purchased from Merck Millipore (Darmstadt, Germany). The anti-SARS-CoV-2 N protein antibody (C524) (catalog no. 3CV4) and recombinant SARS-CoV-2 nucleoprotein (catalog no. 8COV3) were purchased from HyTest (Turku, Finland). The biotinylated anti-SARS-CoV-2 N protein antibody (catalog no. NUN-S47L8) was purchased from ACROBiosystems (Newark, DE, USA). Luria–Bertani (LB) broth was prepared by dissolving 3 g of yeast extract, 6 g of tryptone, and 5 g of NaCl in 600 mL of double-distilled water (ddH_2_O). Phosphate-buffered saline (PBS) was prepared by dissolving 8 g of NaCl, 0.2 g of KCl, 0.24 g of KH_2_PO_4_, and 1.42 g of Na_2_HPO_4_ in 1 L of ddH_2_O, and the pH was adjusted to 7.4.

### 2.2. Preparation of Antibody-Conjugated AuNP Probes

Antibody-conjugated AuNP probes were prepared using a commercial AuNP conjugation kit (Easyget Life Sciences, New Taipei City, Taiwan) according to the manufacturer’s instructions. AuNPs were conjugated with an anti-SARS-CoV-2 N protein antibody (C524) (3CV4, HyTest, Turku, Finland) to prepare the AuNP-Ab probes used in the proposed assay. Separately, AuNPs were conjugated with a biotinylated anti-SARS-CoV-2 N protein antibody (NUN-S47L8, ACROBiosystems) to prepare the biotinylated antibody-conjugated AuNP probes (AuNP-Bio-Ab probes) used in the *E. coli*-scaffold-free dual-AuNP comparison assay. Typically, 37.5 μL of 0.2 M potassium carbonate (K_2_CO_3_) solution was added to 12.5 mL of the AuNP suspension. The mixture was stirred at room temperature for 15 min. Subsequently, 35 μL of the corresponding antibody solution (1 μg/μL) was added, and the mixture was stirred for an additional 15 min at room temperature to allow adsorption of the antibody onto the AuNP surface. Thereafter, 250 μL of blocking buffer was added, followed by stirring for 5 min at room temperature. The resulting suspension was passed through a 0.45 μm syringe filter and transferred to a 50 mL centrifuge tube. The antibody-conjugated AuNPs were collected by centrifugation at 10,000× *g* for 1 h at 4 °C. After removal of the supernatant, the pellet was gently resuspended by pipetting and transferred to a microcentrifuge tube. The suspension was then centrifuged at 8000× *g* for 30 min at 4 °C. Following removal of the supernatant, the final pellet was resuspended in 225 μL of the resuspension buffer provided with the kit. The resulting AuNP-Ab and AuNP-Bio-Ab probe suspensions were stored at 4 °C until use.

### 2.3. Salt-Induced AuNP Aggregation Assay

The colloidal stability of the AuNP-Ab probe was evaluated using a salt-induced aggregation assay. Before testing, the bare AuNP and AuNP-Ab probe suspensions were diluted with deionized water to obtain an identical optical density at their respective peak wavelengths. Each suspension was then evaluated under untreated and salt-challenged conditions. For salt challenge, 100 μL of the bare AuNP or AuNP-Ab probe suspension was mixed with an equal volume of a 10% (*w*/*v*) NaCl solution and incubated at room temperature for 10 min. The corresponding suspensions without NaCl treatment were processed in parallel as controls. Color changes were examined through visual inspection, and UV-Vis absorbance spectra were recorded over the wavelength range of 400–700 nm using a double-beam UV-Vis spectrophotometer (Model UV-1900i, Shimadzu Corp., Kyoto, Japan) equipped with a 10 mm path length quartz cuvette. Colloidal stability was evaluated by comparing the visual appearance and UV-Vis absorption spectra of the NaCl-treated samples with those of the corresponding untreated controls.

### 2.4. Preparation of Streptavidin-Displaying E. coli

*E. coli* MG1655 was used as the host strain for generating streptavidin-displaying bacterial probes. Construction of the pH-SA-eGFP plasmid, preparation of competent cells, and plasmid transformation were performed as described previously [37]. Briefly, 10 μL of the transformed MG1655/pH-SA-eGFP culture was inoculated into 5 mL of LB broth supplemented with 30 μg/mL chloramphenicol and incubated at 37 °C for approximately 16 h. The resulting overnight culture was then diluted into 25 mL of fresh LB broth containing 30 μg/mL chloramphenicol to an initial optical density at 600 nm (OD_600_) of 0.05 and cultured at 37 °C. When the culture reached an OD_600_ of approximately 0.1, L-arabinose was added to a final concentration of 0.02% (*w*/*v*). When the culture reached an OD_600_ of approximately 1.0, IPTG was added to a final concentration of 0.1 mM. The culture was then incubated for an additional 16–18 h at 37 °C. The bacterial cells were harvested through centrifugation at 3000× *g* for 15 min at 4 °C, washed twice with PBS (pH 7.4), and resuspended in PBS for subsequent use.

### 2.5. Preparation of Biotinylated Antibody-Functionalized Streptavidin-Displaying E. coli Probes

One milliliter of the induced MG1655/pH-SA-eGFP suspension adjusted to an OD_600_ of 1.0 was collected, and the bacterial cells were harvested through centrifugation. The cell pellet was resuspended in 50 μL of PBS (pH 7.4), and 10 μL of biotinylated anti-SARS-CoV-2 N protein antibody (NUN-S47L8, ACROBiosystems; 200 ng/μL) was added. The mixture was incubated at 4 °C for 2 h to allow the biotinylated antibody to bind to streptavidin displayed on the bacterial surface. The bacterial cells were then collected through centrifugation at 12,000× *g* for 3 min at 4 °C and washed twice with PBS to remove any unbound antibody. The resulting cell pellet was resuspended in 100 μL of PBS (pH 7.4). The prepared biotinylated antibody-functionalized, streptavidin-displaying *E. coli* probes (*E. coli*-SA/Bio-Ab probes) were stored at 4 °C until use.

### 2.6. Nitrocellulose Membrane-Based Antigen Capture Assay

A nitrocellulose membrane-based antigen capture assay was performed to evaluate the SARS-CoV-2 N protein-binding activity of the *E. coli*-SA/Bio-Ab probes. Briefly, 10 µL of recombinant SARS-CoV-2 N protein (8COV3, HyTest; 10^−11^ mol) was spotted onto a designated region of the nitrocellulose membrane and air-dried overnight at room temperature to allow for protein immobilization. The membrane was then blocked with 3% (*w*/*v*) BSA in PBS for 1 h at room temperature. After blocking, the *E. coli*-SA/Bio-Ab probe suspension was applied to the membrane and allowed to migrate across the antigen-spotted region. Unbound bacterial probes were removed by gently washing the membrane with ddH_2_O. The membrane was briefly air-dried and examined under a fluorescence microscope (DM2500; Leica Microsystems, Wetzlar, Germany).

### 2.7. Storage Stability of Functionalized Probes

The storage stability of the prepared probes was evaluated over a 15-day period. The AuNP-Ab and *E. coli*-SA/Bio-Ab probe suspensions were stored at 4 °C in the dark and analyzed daily. The stability of the AuNP-Ab probes was examined by measuring absorbance at 530 nm, whereas the stability of the *E. coli*-SA/Bio-Ab probes was evaluated by measuring eGFP fluorescence intensity at excitation and emission wavelengths of 485 and 520 nm, respectively, using a multi-mode microplate reader (Synergy H1, BioTek Instruments, Winooski, VT, USA). Before each measurement, the probe suspensions were gently mixed to ensure a homogeneous suspension. The absorbance and fluorescence intensity measured at each time point were compared with the corresponding initial values measured on day 1.

### 2.8. Colorimetric Assay Procedures

Serial dilutions of recombinant SARS-CoV-2 N protein were prepared in PBS (pH 7.4) before the assay. For each reaction, 60 μL of the AuNP-Ab probe suspension was mixed with 10 μL of the recombinant SARS-CoV-2 N protein solution in a sterile 1.5 mL microcentrifuge tube and incubated statically at room temperature (25 ± 1 °C) for 10 min. Subsequently, 30 μL of the *E. coli*-SA/Bio-Ab probe suspension was added, and the colorimetric reaction was allowed to proceed at room temperature for up to 20 min. For comparison, 30 μL of the AuNP-Bio-Ab probe suspension was used in place of the *E. coli*-SA/Bio-Ab probe. Color changes were evaluated through visual inspection using the no-antigen reaction as the negative control, and the corresponding UV-Vis absorbance spectra were recorded.

### 2.9. Data Analysis of Colorimetric Responses

UV-Vis absorbance spectra obtained from the colorimetric assay were used for signal analysis. For each sample, the absorbance values at 530 and 560 nm were obtained from the spectra and designated as A_530_ and A_560_, respectively. The absorbance ratio was calculated as A_560_/A_530_. To correct for background signal, the background-corrected ratio (ΔRatio) was calculated by subtracting the A_560_/A_530_ ratio of the no-target control from that of each sample as follows:

ΔRatio = (A_560_/A_530_), sample − (A_560_/A_530_), no-target control;

The resulting ΔRatio values were used to compare colorimetric responses generated by different concentrations of recombinant SARS-CoV-2 N protein.

## 3. Results

### 3.1. Assay Design and Detection Principle

**Figure 1:**
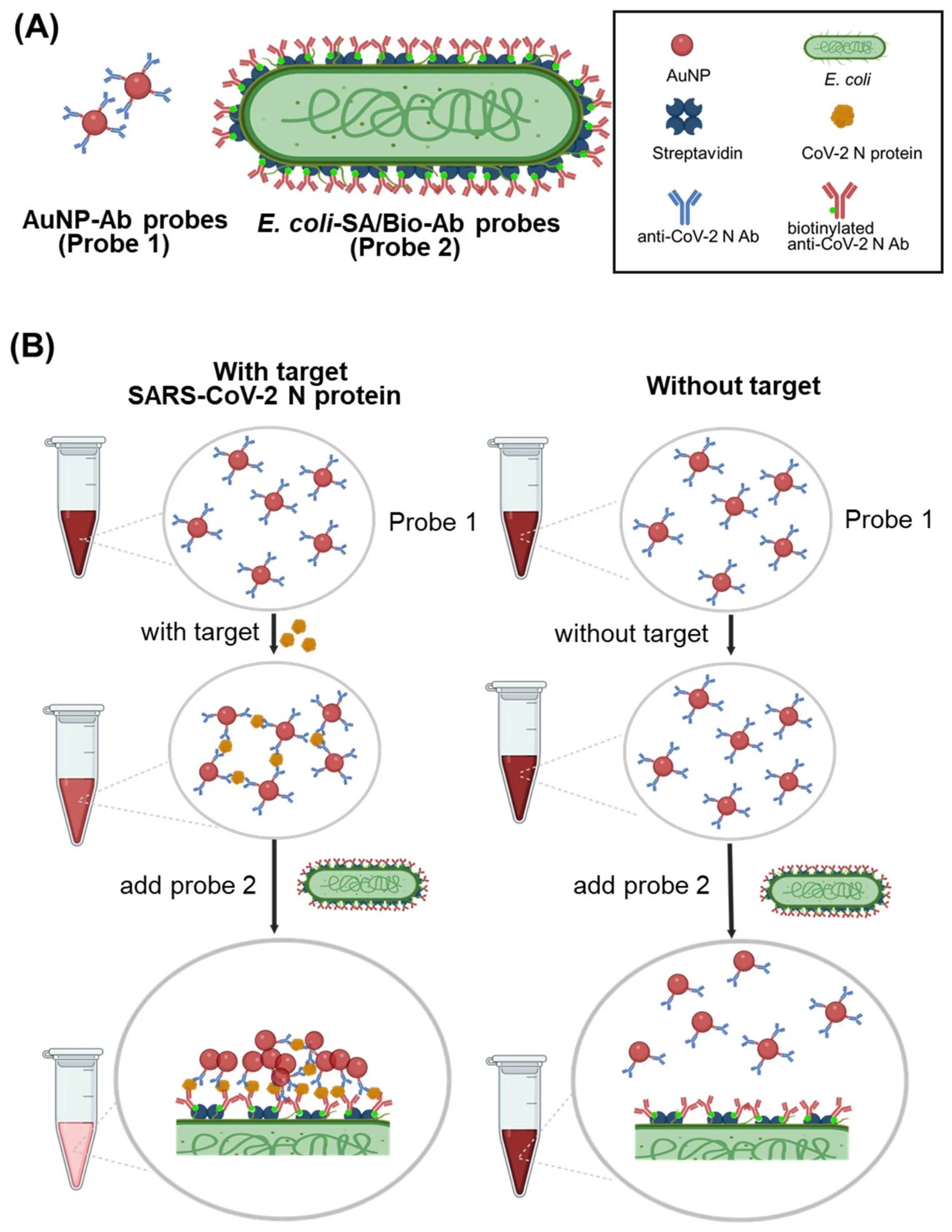
Schematic of the proposed bio-nano colorimetric biosensing platform, evaluated using the SARS-CoV-2 nucleocapsid protein as a representative biomolecular model target. (**A**) Structural components of the plasmonic gold nanoparticle-antibody (AuNP-Ab) signal probes and the engineered *E. coli*-streptavidin/biotinylated antibody (*E. coli*-SA/Bio-Ab) modular capture scaffolds. (**B**) Target-mediated localized plasmonic coupling and colorimetric signal transition in the presence or absence of the model antigen. The schematic was created using the BioRender online platform (BioRender.com).

The design and detection principle of the proposed bio-nano colorimetric immunoassay are illustrated in Figure 1. The proposed solution-phase immunoassay for SARS-CoV-2 N protein detection consists of two functional probe types (Figure 1A): (i) anti-SARS-CoV-2 N protein antibody-conjugated AuNP probes (AuNP-Ab probes), which function as target recognition and colorimetric signal probes, and (ii) biotinylated anti-SARS-CoV-2 N protein antibody-functionalized streptavidin-displaying *E. coli* probes (*E. coli*-SA/Bio-Ab probes), which function as target recognition bio-scaffolds that promote localized AuNP enrichment and colorimetric signal amplification.

The *E. coli*-SA/Bio-Ab probes were prepared using the previously established streptavidin-displaying *E. coli* platform [37]. Briefly, *E. coli* MG1655 was transformed with a plasmid encoding an Lpp-OmpA-streptavidin fusion protein, enabling inducible display of streptavidin on the bacterial surface. Biotinylated anti-SARS-CoV-2 N protein antibodies were then coupled to the surface-displayed streptavidin through the biotin-streptavidin interaction. Because streptavidin contains four biotin-binding sites [33], the surface-displayed streptavidin molecule can provide multiple anchoring sites for biotinylated antibodies, thereby generating the *E. coli*-SA/Bio-Ab probes.

The target-induced colorimetric response is illustrated in Figure 1B. During the assay, the sample was first incubated with the AuNP-Ab probes, and this was followed by the addition of the *E. coli*-SA/Bio-Ab probes. In the presence of SARS-CoV-2 N protein, sequential binding of the antigen to the two probes formed target-mediated bridges, thereby recruiting the antigen-bound AuNP-Ab probes to the bacterial scaffold. This localized accumulation of AuNPs reduces interparticle distance and promotes AuNP clustering, thereby enhancing plasmonic coupling and inducing a visible color change from red to pale-colorless. In the absence of the target antigen, the AuNP-Ab probes remain dispersed in solution, and the suspension retains its characteristic red color. These target-dependent color changes were also reflected in the UV-Vis absorption spectra, providing an objective and quantitative measure of the colorimetric response. Accordingly, the assay was designed to convert antigen-mediated, scaffold-localized AuNP assembly into both a visually observable color change and a quantitative absorbance-based signal.

### 3.2. Characterization and Stability Assessment of the Functional Assay Probes

**Figure 2:**
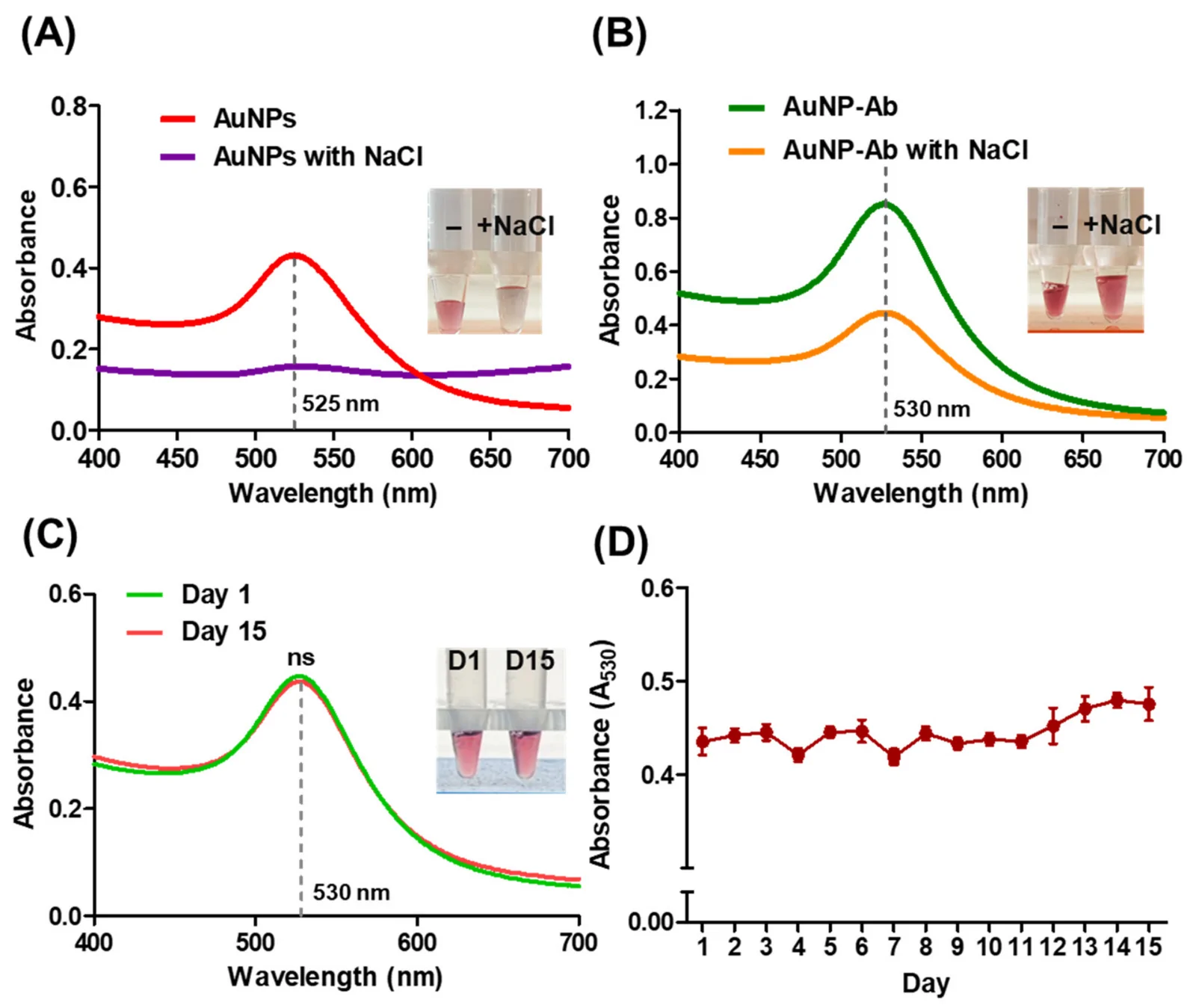
Characterization and colloidal stability assessment of the functionalized sensing probes. (**A**,**B**) Salt-induced aggregation assay for evaluating charge-shielding resistance of (**A**) citrate-capped bare AuNPs and (**B**) antibody-functionalized AuNP (AuNP-Ab) probes. Ultraviolet-visible (UV-Vis) absorbance spectra and corresponding solution photographs (insets) before and after NaCl addition are shown. (**C**,**D**) Low-temperature (4 °C) storage stability of the AuNP-Ab probes over a 15-day evaluation period, showing (**C**) UV-Vis absorbance spectra on days 1 and 15 with representative visual images (inset), and (**D**) longitudinal tracking of daily absorbance values at 530 nm (A_530_) from days 1 to 15. Data are presented as the mean ± standard deviation (n = 3).

To assess antibody functionalization on the AuNP surface, the resistance of the AuNP-Ab probes to salt-induced aggregation was evaluated by comparing the UV-Vis absorbance spectra before and after NaCl addition. Before NaCl treatment, the bare AuNPs exhibited a characteristic absorbance peak at approximately 525 nm, whereas the main absorbance peak of the AuNP-Ab probes was observed at approximately 530 nm. After NaCl addition, the bare AuNPs underwent a pronounced color change accompanied by a marked reduction in the characteristic absorbance peak and a flattened spectral profile (Figure 2A). By contrast, the AuNP-Ab probes retained their characteristic red color, and the absorbance peak at approximately 530 nm remained clearly detectable after NaCl addition (Figure 2B). The aggregation of bare AuNPs under high ionic strength (10% NaCl) is attributed to the screening of electrostatic repulsion between surface-adsorbed citrate ions. In contrast, the robust colloidal stability of AuNP-Ab probes is governed by electrosteric stabilization. The conjugated antibody molecules form a protective macromolecular layer that provides both steric hindrance and a hydration layer, preventing the metallic cores from approaching within the van der Waals attractive regime [38,39]. Furthermore, the storage stability of the AuNP-Ab probes was evaluated. During storage at 4 °C, no discernible colorimetric transition was observed between days 1 and 15, and the UV-Vis spectra obtained at these two time points were highly similar (Figure 2C). Consistent with these observations, the absorbance at 530 nm remained relatively stable throughout the 15-day storage period (Figure 2D). Overall, these results support the successful conjugation of antibodies to the AuNP surface and demonstrate that the resulting AuNP-Ab probes exhibited robust salt tolerance and colloidal stability during storage.

**Figure 3:**
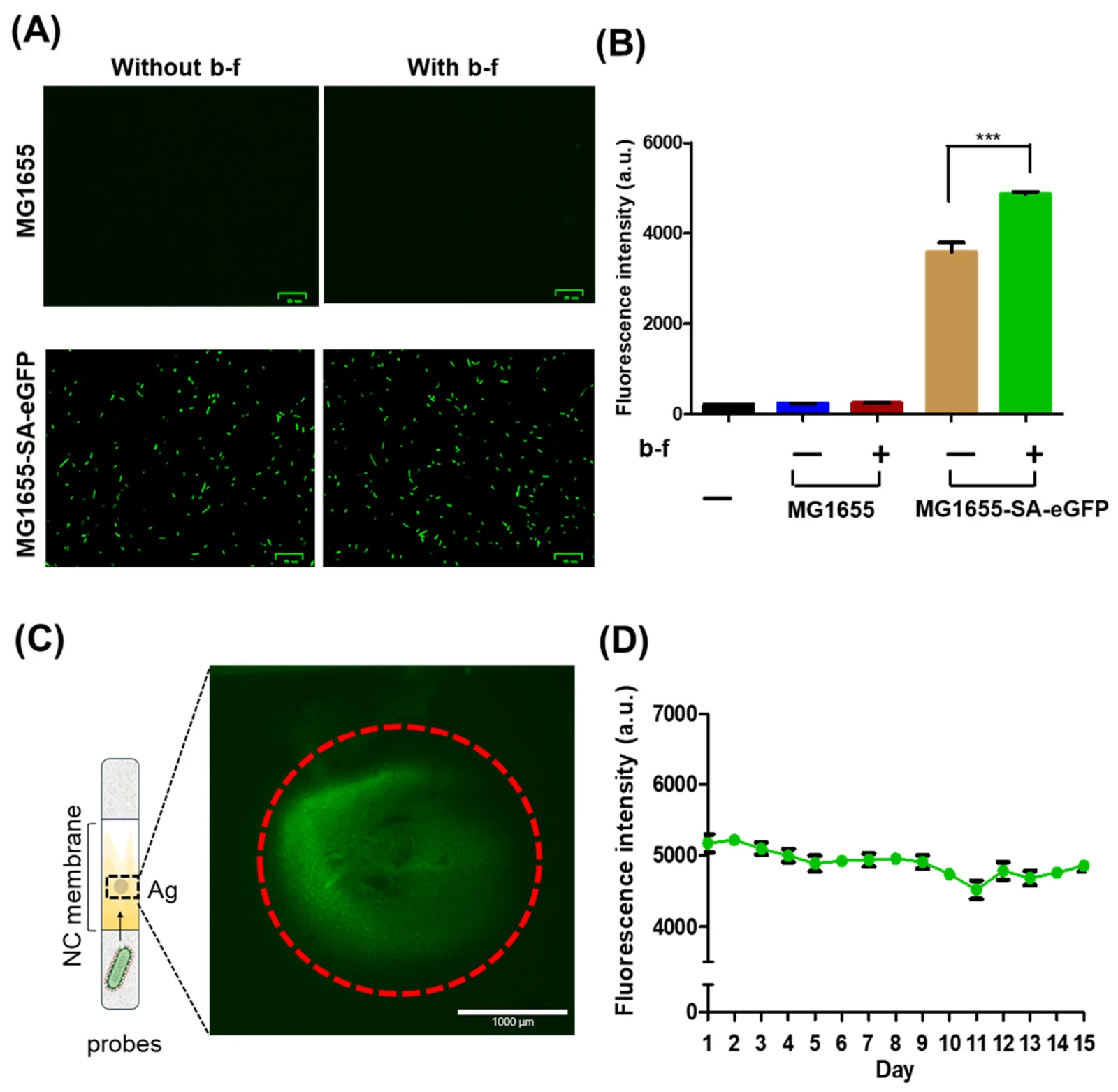
Surface functionalization, ligand-binding validation, and storage stability of the *E. coli*-SA/Bio-Ab probes. (**A**,**B**) Validation of streptavidin surface display: (**A**) Representative fluorescence microscopy images of wild-type MG1655 and engineered MG1655 streptavidin-displaying and enhanced green fluorescent protein (eGFP)-expressing cells in the presence or absence of biotin-fluorescein (b-F) conjugates; (**B**) Statistical quantification of cell-bound b-F fluorescence intensity obtained from three independent experiments. Data are presented as the mean ± standard deviation (*** *p* < 0.001). (**C**) Representative fluorescence microscopy images from the nitrocellulose membrane antigen-capture assay, demonstrating the target-binding bioactivity of the *E. coli*-SA/Bio-Ab probes. (**D**) Longitudinal storage stability of the *E. coli*-SA/Bio-Ab probes over a 15-day evaluation period at 4 °C, monitored via the retention of intracellular eGFP reporter fluorescence. Data are presented as the mean ± standard deviation (n = 3).

Before preparing the *E. coli*-SA/Bio-Ab probes, we evaluated the surface display and biotin-binding activity of streptavidin in engineered *E. coli* MG1655. MG1655 was transformed with the previously reported pH-SA-eGFP plasmid [37]. The transformed cells displayed streptavidin on the bacterial surface and expressed enhanced green fluorescent protein (eGFP) intracellularly, allowing for cell visualization and subsequent monitoring of probe stability. To evaluate the functionality of the surface-displayed streptavidin, engineered and non-transformed MG1655 cells were incubated with biotin-fluorescein (b-F). As presented in Figure 3A, non-transformed MG1655 cells exhibited negligible green fluorescence regardless of b-F treatment, indicating minimal intrinsic fluorescence and negligible nonspecific b-F binding. By contrast, engineered MG1655 cells exhibited pronounced green fluorescence even in the absence of b-F, reflecting intracellular eGFP expression. After b-F incubation, cell-associated fluorescence further increased. Quantitative fluorescence analysis confirmed that b-F-treated engineered MG1655 cells exhibited significantly higher fluorescence intensity than both untreated engineered cells and non-transformed control cells (Figure 3B). These findings are consistent with our previous flow cytometry results demonstrating increased biotin-fluorescein binding by MG1655/pH-SA-eGFP cells [37]. Overall, these results demonstrate that streptavidin was successfully displayed on the surface of the engineered MG1655 and retained its biotin-binding activity, supporting its subsequent coupling with biotinylated anti-SARS-CoV-2 N protein antibodies.

After confirming the surface display and biotin-binding activity of streptavidin, we evaluated the antigen-binding activity of the resulting *E. coli*-SA/Bio-Ab probes using a membrane-based antigen capture assay. This membrane-based experiment was used solely as a functional validation of the antigen-binding activity of the bacterial probes. The probes were applied to a nitrocellulose membrane containing recombinant SARS-CoV-2 N protein immobilized at defined locations, and antigen binding was assessed based on probe retention at these locations. As presented in Figure 3C, localized green fluorescence was observed at the SARS-CoV-2 N protein-coated regions, indicating that the probes were captured through antigen–antibody binding. The storage stability of the *E. coli*-SA/Bio-Ab probes was also evaluated at 4 °C for 15 days. The eGFP-derived fluorescence intensity remained stable throughout the storage period, with no marked decrease in signal intensity (Figure 3D). Collectively, these results demonstrate that the prepared *E. coli*-SA/Bio-Ab probes retained their functional activity and remained stable throughout the evaluated storage period.

### 3.3. Optimization of the Testing Procedures

For the proposed solution-phase sandwich-like colorimetric assay, signal generation requires efficient target-mediated assembly of the AuNP-Ab probes and the *E. coli*-SA/Bio-Ab probes. Because the two probe types substantially differ in size, composition, and function, the sequence of probe addition may affect antigen–antibody binding, probe accessibility, and subsequent AuNP clustering. Therefore, we first evaluated the effect of the probe addition sequence to establish an efficient assay workflow. Two assay formats were compared. In the first format, the sample was incubated with the AuNP-Ab probes before the addition of the *E. coli*-SA/Bio-Ab probes. In the second format, the sample was first incubated with the *E. coli*-SA/Bio-Ab probes before the addition of the AuNP-Ab probes.

**Figure 4:**
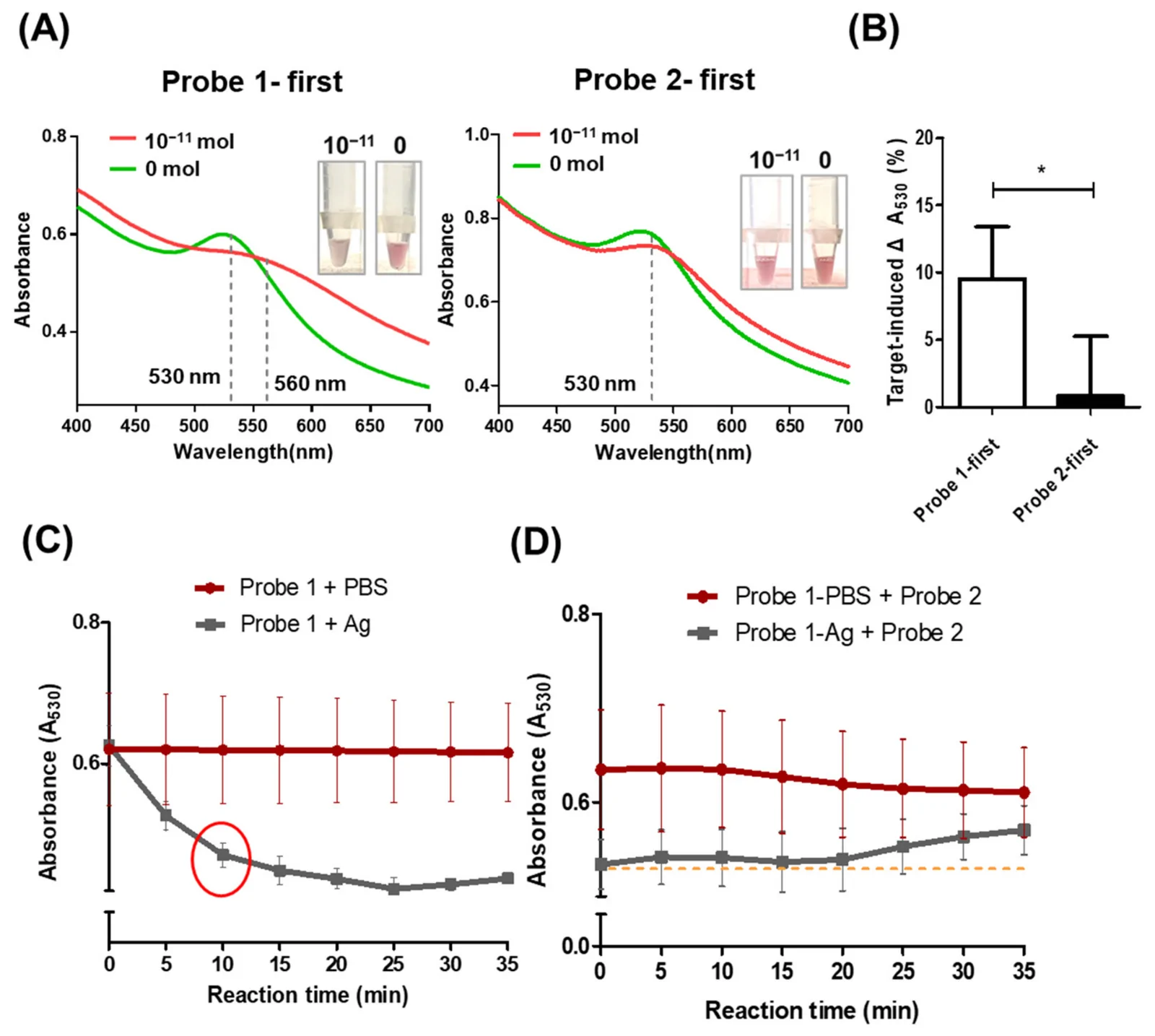
Optimization of the reaction conditions for the proposed biosensing platform. AuNP-Ab and *E. coli*-SA/Bio-Ab probes are denoted as Probe 1 and Probe 2, respectively. (**A**) Optimization of the probe addition sequence in the absence and presence of the model analyte, evaluating the alleviation of steric hindrance constraints. UV-Vis absorbance spectra and corresponding solution photographs (insets) are shown for both the “Probe 1-first” and “Probe 2-first” schemes. For (**B**–**D**), data are presented as the mean ± standard deviation (n = 3). (**B**) Comparative analysis of the target-induced relative decrease in absorbance at 530 nm (ΔA_530_) between the two incubation sequences. The percentage decrease (A_530_) was calculated using the formula: [(A_530_, no target − A_530_, target)/A_530_, no target] × 100%. * *p* < 0.05. (**C**) Kinetic optimization of the primary incubation duration between Probe 1 and the target antigen. (**D**) Kinetic optimization of the secondary incubation duration following the recruitment of Probe 2 onto the preformed Probe 1-antigen complex. The amount of SARS-CoV-2 N protein used in the antigen-containing groups was 10^−11^ mol.

To provide clear visual and spectroscopic responses during assay optimization, a model target amount of 10^−11^ mol of recombinant SARS-CoV-2 N protein was selected based on preliminary screening of the assay conditions. This target amount provided a distinct response while avoiding premature signal saturation, allowing differences among the tested reaction conditions to be more clearly evaluated. As presented in Figure 4A, when the sample was first incubated with the AuNP-Ab probes and then with the *E. coli*-SA/Bio-Ab probes, the presence of SARS-CoV-2 N protein produced a visually detectable color change relative to the antigen-free control. The corresponding UV-Vis absorbance spectra showed a clear change in spectral profile, with the characteristic peak at approximately 530 nm becoming less distinct compared with that of the antigen-free control. By contrast, when the *E. coli*-SA/Bio-Ab probes were added before the AuNP-Ab probes, the target-positive sample also exhibited a visible color change relative to the antigen-free control. However, the color change was less pronounced than that observed when the AuNP-Ab probes were added first. Quantitative analysis of the absorbance at 530 nm further demonstrated that the AuNP-Ab-first format resulted in a greater target-induced change in absorbance than the reverse probe-addition sequence (Figure 4B). Consistent with these findings, additional UV-Vis spectra obtained with different amounts of SARS-CoV-2 N protein (10^−13^–10^−11^ mol) showed spectral changes across the tested amounts in the AuNP-Ab-first format, whereas the spectra obtained using the reverse probe-addition sequence were more closely clustered (Supplementary Figure S1). Overall, these results demonstrate that the AuNP-Ab-first format provided a stronger colorimetric response, and it was therefore selected for subsequent experiments.

The reaction time for the selected AuNP-Ab-first format was then optimized in two sequential steps. Initially, the kinetics of the probe-target interaction was investigated by incubating the AuNP-Ab probes with the SARS-CoV-2 N protein across varying durations to establish the optimal binding equilibrium. As presented in Figure 4C, the absorbance of the AuNP-Ab/N protein mixture at 530 nm gradually decreased during the initial incubation period and reached a relatively stable level within 10 min. No appreciable change was observed from 10 to 35 min. Therefore, 10 min was selected as the incubation time for the first-step AuNP-Ab/N protein reaction. In the second step, the *E. coli*-SA/Bio-Ab probes were added to the preformed AuNP-Ab/N protein mixture, and the reaction was monitored for different incubation times. The absorbance at 530 nm remained relatively stable during the first 20 min after the addition of the *E. coli*-SA/Bio-Ab probes but exhibited a slight upward trend after 25 min (Figure 4D). Therefore, the second-step reaction time was limited to 20 min. On the basis of these results, the optimized assay consisted of a 10 min incubation of the sample with the AuNP-Ab probes, followed by incubation of the *E. coli*-SA/Bio-Ab probes for up to 20 min, resulting in a total assay time of no more than 30 min.

### 3.4. Sensitivity of the Proposed Assay with AuNPs as Colorimetric Signals

**Figure 5:**
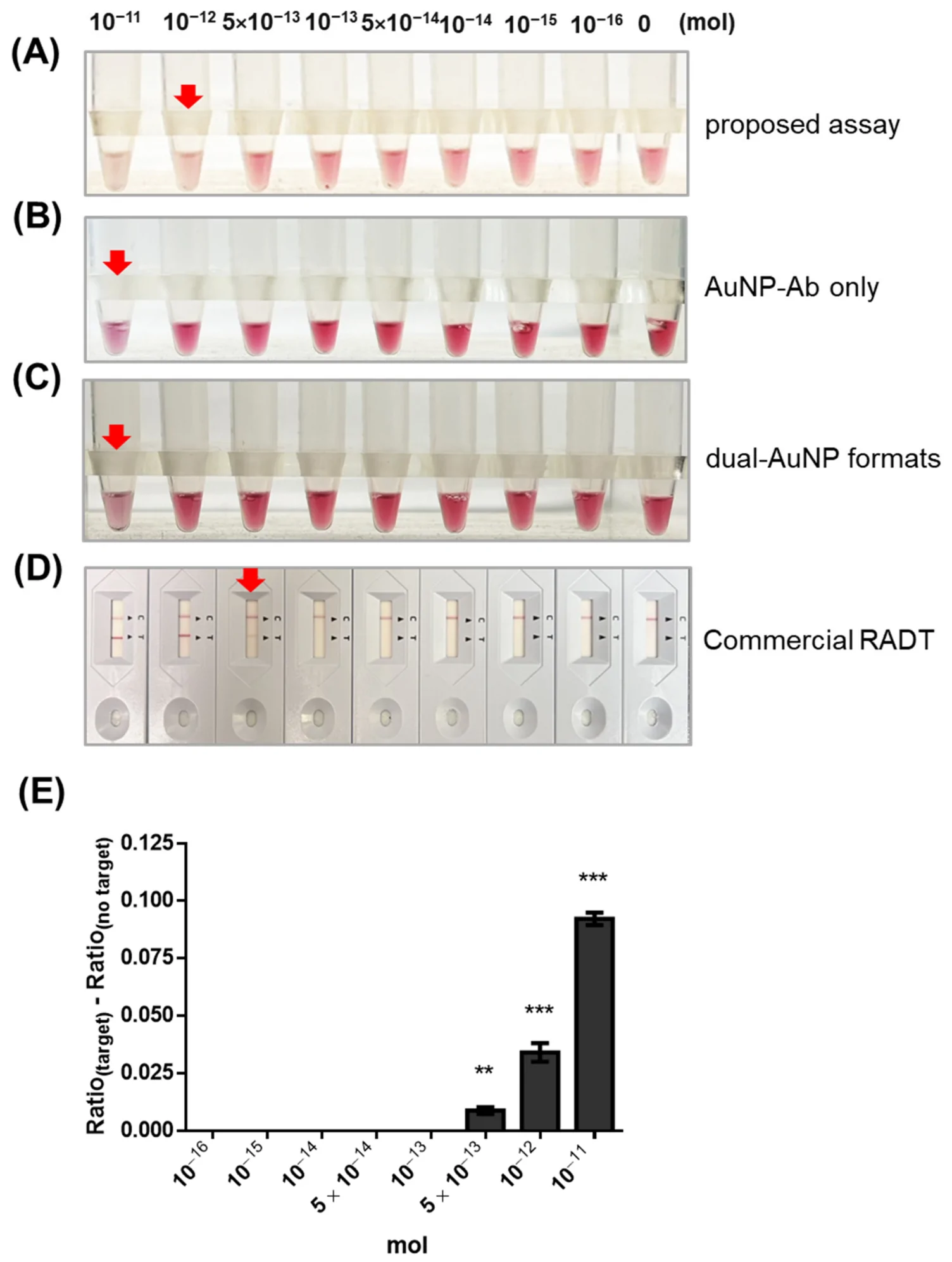
Comparative analytical evaluation of the proposed colorimetric biosensing platform. Comparison of the model analyte (recombinant SARS-CoV-2 N protein) detection using: (**A**) the proposed bacterial-scaffold-mediated assay; (**B**) the AuNP-Ab signal probe-only control format; (**C**) the *E. coli*-scaffold-free dual-AuNP control format comprising AuNP-Ab and AuNP-Bio-Ab probes; and (**D**) a commercial rapid antigen detection test (RADT; Roche SARS-CoV-2 Antigen Self-Test Nasal). Red arrows indicate the lowest target amount at which a visually discernible response was observed for each assay format. (**E**) Absorbance-based quantitative evaluation of the colorimetric response generated by the proposed platform. The *x*-axis indicates the absolute amount of the model N protein, and the *y*-axis indicates the background-corrected ratio difference, calculated as: ΔRatio = (A_560_/A_530_), target − (A_560_/A_530_), no target. Data are presented as the mean ± standard deviation (** *p* < 0.01, *** *p* < 0.001).

The detection performance of the proposed assay was evaluated using recombinant SARS-CoV-2 N protein. Figure 5A–D presents representative visual results obtained using the proposed assay and the comparison assay formats tested in parallel. In the proposed assay, the no-target control retained the characteristic red color of the AuNP suspension. As the amount of N protein increased, enhanced target-mediated bridging between the AuNP-Ab probes and the *E. coli*-SA/Bio-Ab probes promoted AuNP assembly, resulting in a progressive decrease in red color intensity. The lowest amount of N protein that produced a visually discernible color change relative to the no-target control was 10^−12^ mol (Figure 5A), demonstrating the feasibility of the proposed assay for instrument-free visual detection of SARS-CoV-2 N protein.

To evaluate the contribution of the engineered *E. coli* scaffold to visual signal enhancement, the proposed assay was compared with two simplified assay formats that used the same anti-SARS-CoV-2 N protein antibody system. The first format used solely the AuNP-Ab signal probe. In the second format, the AuNP-Ab signal probe was retained, whereas the *E. coli*-SA/Bio-Ab probe was replaced with biotinylated antibody-conjugated AuNPs (AuNP-Bio-Ab probes), resulting in an *E. coli*-scaffold-free dual-AuNP format. In both simplified formats, the lowest amount of N protein that produced a visually discernible color change relative to the no-target control was 10^−11^ mol (Figure 5B,C), indicating that the proposed assay achieved an approximately 10-fold improvement in visual detection sensitivity. Notably, the single-probe format and the *E. coli*-scaffold-free dual-AuNP format exhibited comparable visual detection sensitivity, suggesting that the addition of a second antibody-conjugated AuNP probe alone did not effectively improve signal amplification. By contrast, the engineered *E. coli* scaffold may enhance the colorimetric response by presenting biotinylated antibodies on the bacterial surface through surface-displayed streptavidin, thereby facilitating the antigen-mediated recruitment of AuNP-Ab probes to the bacterial scaffold. This localized assembly may increase the local concentration of AuNPs and promote nanoparticle clustering, resulting in a more pronounced colorimetric response. Specifically, the dramatic 10-fold enhancement in visual sensitivity (LOD of 10^−12^ mol) achieved by the *E. coli* scaffold is consistent with a contribution from scaffold-mediated localized plasmonic coupling. When AuNP-Ab probes are recruited onto the micrometer-scale, three-dimensional cellular interface, the target antigen acts as a molecular bridge, favoring the recruitment of AuNPs into close proximity. As the interparticle distance (d) decreases relative to the nanoparticle core diameter (D), near-field electromagnetic interactions between adjacent AuNPs become increasingly pronounced, leading to interparticle plasmon coupling and a red-shift in the plasmon resonance [40,41]. These plasmonic changes can translate subtle molecularly induced nanoparticle assembly into a readily observable visual color change.

The proposed assay was evaluated alongside a commercial SARS-CoV-2 rapid antigen test (Roche SARS-CoV-2 Antigen Self-Test Nasal). The commercial test showed a visually detectable response at 5 × 10^−13^ mol of N protein (Figure 5D), serving as a qualitative visual reference point under our tested conditions. Rather than a quantitative analytical comparison, this parallel evaluation was intended to contextualize the visual readability of our platform’s naked-eye colorimetric response. Overall, these results support the visual detection capability of the proposed assay, suggesting its potential for further development as an instrument-free detection platform.

To further evaluate the quantitative colorimetric response of the proposed assay, an absorbance-based analysis was performed. In this assay, the absorbance at 530 nm (A_530_) represents the dispersed state of the AuNP-Ab probes in suspension, whereas the absorbance at 560 nm (A_560_) was selected based on the spectral red-shift induced by the target-mediated plasmonic coupling. Accordingly, the colorimetric response was expressed as the A_560_/A_530_ ratio, and the change in this ratio relative to the no-target control was used to quantify the target-induced optical response. As presented in Figure 5E, the ratio difference remained close to the baseline at lower amounts of N protein but increased as the target amount increased. A statistically significant increase was first observed at 5 × 10^−13^ mol of N protein, with the current system showing a quantitative response over the evaluated range from 5 × 10^−13^ to 10^−11^ mol. The inter-day coefficients of variation (CVs) were 16.10%, 11.77%, and 2.95% at 5 × 10^−13^, 10^−12^, and 10^−11^ mol of N protein, respectively, providing a preliminary indication of the inter-day reproducibility of the proposed assay. While a comprehensive and systematic robustness assessment of analytical variables remains to be performed in future development, the observed salt tolerance of the AuNP-Ab probes and their wet-storage stability provide preliminary support for the operational stability of the assay components under the evaluated laboratory conditions. Overall, these results support the potential of the proposed assay to transition from a primarily visual readout toward quantitative assessment of the colorimetric response.

## 4. Discussion

In this study, a bio-nano colorimetric immunoassay was developed by incorporating streptavidin-displaying *E. coli* into an antibody-functionalized AuNP-based detection system. The engineered bacterial scaffold serves as a multivalent biological interface that promotes target-mediated AuNP assembly and generates a visible colorimetric response. For SARS-CoV-2 N protein detection, the proposed assay achieved a visual detection limit of approximately 10^−12^ mol, whereas the AuNP-Ab probe-only and *E. coli*-scaffold-free dual-AuNP formats achieved detection limits of approximately 10^−11^ mol (Figure 5A–C). These comparative results suggest that the engineered bacterial scaffold substantially contributes to the observed signal amplification. Previous studies have reported that the Lpp-OmpA system enables the surface display of functionally active streptavidin on *E. coli* [36]. Engineered *E. coli* surface-display systems have also been reported to display approximately 10^4^–10^5^ functional molecules per cell [42]. In a related autodisplayed streptavidin system, approximately 1.6 × 10^5^ streptavidin molecules were estimated to be displayed on each *E. coli* cell [43]. Furthermore, biotinylated horseradish peroxidase saturation analysis indicated that engineered *E. coli* exhibited at least a 3-fold greater functional biotin-binding capacity than streptavidin-coated magnetic beads did [43]. Such a high-density streptavidin interface can support dense immobilization of biotinylated capture antibodies, thereby providing multiple binding sites for antigen capture and subsequent recruitment of AuNP-Ab signal probes to the bacterial surface. This multivalent recruitment promotes the localized enrichment of AuNPs within a confined three-dimensional interfacial region while constraining the spatial dispersion of the recruited AuNP-Ab signal probes relative to freely suspended probes. The resulting increase in local AuNP density may bring neighboring nanoparticles into closer proximity, thereby favoring plasmonic coupling and enhancing the visible colorimetric response. Although the present optical, spectroscopic, and control data are consistent with scaffold-mediated AuNP clustering and enhanced plasmonic interactions, the lack of direct morphological characterization remains a limitation of this study, as our current setup does not directly resolve the spatial topography or precise physical dimensions of the surface-associated AuNP assemblies. To obtain such structural information while minimizing sample-preparation artifacts, future studies will focus on integrating advanced direct imaging techniques to further characterize the morphology and cluster size of the scaffold-mediated assembly.

In addition to the high density of antibody presentation enabled by surface-displayed streptavidin, the orientation of biotinylated antibodies on the bacterial scaffold may affect target capture efficiency and subsequent signal amplification. Site-directed biotinylation at the antibody hinge region has been reported to promote oriented antibody immobilization, resulting in improved antigen recognition and immunoassay performance compared with random amine-directed biotinylation [44,45]. Accordingly, incorporating site-directed biotinylation into the *E. coli*-SA/Bio-Ab probes in future studies may lead to more favorable antibody orientation and improve the accessibility of antigen-binding sites. This, in turn, may facilitate more efficient target capture and AuNP recruitment, thereby further enhancing colorimetric sensitivity.

The sequence of probe addition is a crucial consideration in sandwich-like immunoassays because it can affect epitope accessibility, initial probe-target binding, and subsequent target-mediated bridging. In this study, preincubating SARS-CoV-2 N protein with the AuNP-Ab probes before adding the *E. coli*-SA/Bio-Ab probes resulted in a more pronounced colorimetric response than the reverse order of probe addition did (Figure 4A). This order-dependent difference suggests that the formation of AuNP-Ab/N protein complexes during the initial incubation step facilitates their subsequent binding to the *E. coli*-SA/Bio-Ab probes. By contrast, when the *E. coli*-SA/Bio-Ab probes were added first, the relatively large size of the bacterial cells may impose steric hindrance [46,47], limiting the access of the subsequently added AuNP-Ab probes to the N protein antigen. In addition, the structural features of the *E. coli* MG1655 surface, including lipopolysaccharides and fimbria-associated components [48,49], may increase local steric hindrance and further restrict access of the AuNP-Ab probes to the surface-bound antigen. Overall, these spatial and steric constraints may explain the less pronounced colorimetric response observed when the *E. coli*-SA/Bio-Ab probes were added before the AuNP-Ab probes. These findings suggest that the sequence of probe addition affects the efficiency of scaffold-guided AuNP assembly and, consequently, the performance of the proposed assay.

To evaluate its potential for further development as a practical SARS-CoV-2 rapid antigen test, the proposed assay was assessed in parallel with a commercially available rapid antigen test using the same amounts of recombinant SARS-CoV-2 N protein. The proposed assay achieved a naked-eye LOD of approximately 10^−12^ mol, whereas the commercial test achieved an LOD of approximately 5 × 10^−13^ mol (Figure 5A,D). On the basis of the assay volume used in this study, an LOD of 10^−12^ mol corresponded to an estimated concentration of approximately 460 ng/mL. This concentration is comparable to the reported LOD range of 438–639 ng/mL for a previously developed screened Fv antibody-based one-step immunoassay for recombinant SARS-CoV-2 N protein detection [50]. Overall, the analytical sensitivity of the proposed assay was comparable to that reported for a previously developed recombinant SARS-CoV-2 antigen immunoassay. Notably, this level of analytical sensitivity was achieved using a naked-eye colorimetric readout without requiring additional instrumentation, complex surface fabrication, or complicated assay workflows. These findings support the feasibility of the proposed assay as a laboratory-stage prototype. Physicochemically, the maintained colloidal stability of the AuNP-Ab probes under high-salt challenge (Figure 2B) suggests that the antibody-functionalized surface may contribute to the overall electrosteric stabilization of the particles. From a Derjaguin–Landau–Verwey–Overbeek (DLVO)-based perspective [51,52], this observation suggests that the signal probes may retain colloidal stability under elevated ionic-strength conditions relevant to biological matrices, such as saliva or nasal secretions. In addition, the exceptionally high affinity of the biotin–streptavidin interaction (K_d_ approximately 10^−15^ M) supports the potential for stable anchoring of the biotinylated antibody on the bacterial scaffold in the presence of background proteins. Nevertheless, validation in representative biological matrices and clinical specimens remains necessary for further translation into practical diagnostic use.

In the context of practical implementation, the proposed bacterial scaffold may offer potential advantages in probe fabrication compared with conventional bead- or planar surface-based platforms. Although preparation of the *E. coli*-SA/Bio-Ab probes involves bacterial cultivation, induction, and antibody coupling, the bacterial scaffold itself can be produced at relatively low cost and readily scaled up. Direct surface expression of streptavidin also eliminates the need for separate purification steps or additional carrier functionalization. In comparison, commercial streptavidin-coated beads represent a recurring consumable cost, whereas the cost of planar antibody-immobilization platforms varies with substrate materials, surface functionalization, and antibody consumption. In addition to these cost considerations, batch-to-batch reproducibility is also important for practical deployment of the bacterial platform, as it may be affected by the consistency of bacterial growth, surface streptavidin expression, and antibody coupling efficiency. Furthermore, long-term shelf-life constitutes an additional consideration. Because the bacterial cells function primarily as structural scaffolds for surface biomolecule display rather than as metabolically active components, their viability is not expected to be a critical determinant of probe performance. Nevertheless, prolonged storage in aqueous suspension may affect bacterial membrane integrity and the stability of surface-associated biomolecules. Accordingly, dry-state preservation strategies such as lyophilization may be explored in future development to improve long-term stability.

In summary, this study provides proof of concept for integrating the biotin-streptavidin coupling system with an *E. coli*-AuNP bio-nano assembly to establish a solution-phase colorimetric platform for viral antigen detection. The proposed platform incorporates features relevant to several key principles of the ASSURED framework for point-of-care diagnostics. Its AuNP-based naked-eye colorimetric readout reduces reliance on analytical instrumentation, whereas the solution-phase format simplifies the assay workflow by eliminating the need for complex surface fabrication and reducing the transport limitations associated with membrane-based assays. In addition, the engineered *E. coli* scaffold promotes localized AuNP assembly and signal amplification without requiring enzymes or complex signal-enhancement procedures. The biotin-streptavidin interface also enables modular coupling of biotinylated recognition elements, supporting the potential adaptation of the platform to different diagnostic targets. Overall, these features demonstrate the feasibility of a visually interpretable and adaptable bio-nano colorimetric detection platform with potential for further development as a practical point-of-care antigen assay.

## 5. Conclusions

In this study, we developed a solution-phase bio-nano colorimetric assay that employs streptavidin-displaying *E. coli* as a multifunctional bacterial scaffold to enhance the visual detection of biological targets. Using the SARS-CoV-2 N protein as a representative biomolecular model, the target antigen bridged the *E. coli*-SA/Bio-Ab and AuNP-Ab probes, promoting scaffold-directed, localized plasmonic coupling around the bacterial interface to generate a distinct visual colorimetric response. The assay was completed within 30 min and achieved a naked-eye LOD of approximately 10^−12^ mol, representing a 10-fold improvement in visual detection sensitivity compared with both the AuNP-Ab probe-only and *E. coli*-scaffold-free dual-AuNP formats (LOD of 10^−11^ mol). Notably, the qualitative comparison with a commercial rapid antigen test provided a practical visual benchmark, supporting the feasibility of our assay as an instrument-free platform for decentralized biosensing.

Overall, this study highlights three key features of the proposed platform that underscore its versatility for colorimetric biosensing. First, the solution-phase, target-mediated assembly minimizes the transport limitations and mass-transfer resistance typical of membrane-based lateral flow formats. Second, the engineered *E. coli* scaffold provides a high-capacity, multivalent interface for antigen-mediated recruitment and localized enrichment of AuNP-Ab signal probes, potentially limiting their diffusional freedom and favoring closer proximity between AuNPs conducive to plasmonic coupling, thereby enhancing the optical response without requiring auxiliary enzyme-mediated reactions. Third, the biotin-streptavidin coupling interface offers a highly modular, plug-and-play architecture. By simply swapping the biotinylated recognition ligands (such as antibodies or aptamers), this adaptable platform can be readily extended to detect a wide range of clinically relevant biomarkers, foodborne pathogens, or waterborne toxins, thereby supporting its potential for point-of-care diagnostics and rapid, on-site environmental monitoring applications.

## Data Availability

The original contributions presented in this study are included in the article/Supplementary Material. Further inquiries can be directed to the corresponding authors.

## Supplementary Materials

The following supporting information can be downloaded at: https://www.mdpi.com/article/10.3390/s26185965/s1, Figure S1: UV-Vis absorbance spectra of the sensing system at different amounts of recombinant SARS-CoV-2 N protein with different probe-addition sequences. AuNP-Ab and *E. coli*-SA/Bio-Ab probes are denoted as Probe 1 and Probe 2, respectively. Spectra were obtained with 0, 10^−13^, 10^−12^, and 10^−11^ mol of recombinant SARS-CoV-2 N protein using (A) the Probe 1-first sequence and (B) the Probe 2-first sequence. The dashed lines indicate 530 nm.

## Abbreviations

The following abbreviations are used in this manuscript:

AuNPs	Gold nanoparticles
POCT	Point-of-care testing
SARS-CoV-2	Severe acute respiratory syndrome coronavirus 2
RSV	Respiratory syncytial virus
RT-qPCR	Real-time reverse transcription quantitative polymerase chain reaction
N	Nucleocapsid
K_d_	Dissociation constant
BSA	Bovine serum albumin
ddH_2_O	Double-distilled water
IPTG	Isopropyl β-D-1-thiogalactopyranoside
LB medium	Luria–Bertani medium
PBS	Phosphate-buffered saline
eGFP	Enhanced green fluorescent protein
b-F	Biotin-fluorescein
A_530_	Absorbance at 530 nm
A_560_	Absorbance at 560 nm
DLVO	Derjaguin–Landau–Verwey–Overbeek

## References

[1] Hayden O., Luppa P.B., Min J. (2022). Point-of-care testing-new horizons for cross-sectional technologies and decentralized application strategies. Anal. Bioanal. Chem..

[2] Nichols J.H., Alter D., Chen Y., Isbell T.S., Jacobs E., Moore N., Shajani-Yi Z. (2020). AACC Guidance Document on Management of Point-of-Care Testing. J. Appl. Lab. Med..

[3] Hansen S., Abd El Wahed A. (2020). Point-Of-Care or Point-Of-Need Diagnostic Tests: Time to Change Outbreak Investigation and Pathogen Detection. Trop. Med. Infect. Dis..

[4] Leon-Justel A., Navarro Bustos C., Noval-Padillo J.A., Martin Perez S., Aviles Gomez M.D., Jimenez Valencia N., Garrido Castilla J.M., Diaz Munoz M., Rivera Vizcaino M.A., Alvarez Heredia L. (2025). Point-of-care testing improves care timeliness in the emergency department. A multicenter randomized clinical trial (study POCTUR). Clin. Chem. Lab. Med..

[5] Petrucci S., Costa C., Broyles D., Dikici E., Daunert S., Deo S. (2021). On-site detection of food and waterborne bacteria—Current technologies, challenges, and future directions. Trends Food Sci. Technol..

[6] Nnachi R.C., Sui N., Ke B., Luo Z., Bhalla N., He D., Yang Z. (2022). Biosensors for rapid detection of bacterial pathogens in water, food and environment. Environ. Int..

[7] Mabey D., Peeling R.W., Ustianowski A., Perkins M.D. (2004). Diagnostics for the developing world. Nat. Rev. Microbiol..

[8] Havasi A., Visan S., Cainap C., Cainap S.S., Mihaila A.A., Pop L.A. (2022). Influenza A, Influenza B, and SARS-CoV-2 Similarities and Differences—A Focus on Diagnosis. Front. Microbiol..

[9] Geismar C., Nguyen V., Fragaszy E., Shrotri M., Navaratnam A.M.D., Beale S., Byrne T.E., Fong W.L.E., Yavlinsky A., Kovar J. (2023). Symptom profiles of community cases infected by influenza, RSV, rhinovirus, seasonal coronavirus, and SARS-CoV-2 variants of concern. Sci. Rep..

[10] Binny R.N., Priest P., French N.P., Parry M., Lustig A., Hendy S.C., Maclaren O.J., Ridings K.M., Steyn N., Vattiato G. (2022). Sensitivity of Reverse Transcription Polymerase Chain Reaction Tests for Severe Acute Respiratory Syndrome Coronavirus 2 Through Time. J. Infect. Dis..

[11] Hanson K.E., Azar M.M., Banerjee R., Chou A., Colgrove R.C., Ginocchio C.C., Hayden M.K., Holodiny M., Jain S., Koo S. (2020). Molecular Testing for Acute Respiratory Tract Infections: Clinical and Diagnostic Recommendations From the IDSA’s Diagnostics Committee. Clin. Infect. Dis..

[12] Iliescu F.S., Ionescu A.M., Gogianu L., Simion M., Dediu V., Chifiriuc M.C., Pircalabioru G.G., Iliescu C. (2021). Point-of-Care Testing-The Key in the Battle against SARS-CoV-2 Pandemic. Micromachines.

[13] Frank F., Keen M.M., Rao A., Bassit L., Liu X., Bowers H.B., Patel A.B., Cato M.L., Sullivan J.A., Greenleaf M. (2022). Deep mutational scanning identifies SARS-CoV-2 Nucleocapsid escape mutations of currently available rapid antigen tests. Cell.

[14] Huang R., Cai X., Du J., Lian J., Hui P., Gu M., Li F., Wang J., Chen W. (2022). Bioinspired Plasmonic Nanosensor for on-Site Antimicrobial Susceptibility Testing in Urine Samples. ACS Nano.

[15] Marin M., Nikolic M.V., Vidic J. (2021). Rapid point-of-need detection of bacteria and their toxins in food using gold nanoparticles. Compr. Rev. Food Sci. Food Saf..

[16] Yang S.Z., Liu Q.A., Liu Y.L., Weng G.J., Zhu J., Li J.J. (2021). Recent progress in the optical detection of pathogenic bacteria based on noble metal nanoparticles. Mikrochim. Acta.

[17] Restrepo-Cano V., Garcia-Huertas P., Caraballo-Guzman A., Sanchez-Jimenez M.M., Torres-Lindarte G. (2025). Back to Basics: Unraveling the Fundamentals of Lateral Flow Assays. J. Appl. Lab. Med..

[18] Cho D.G., Yoo H., Lee H., Choi Y.K., Lee M., Ahn D.J., Hong S. (2018). High-Speed Lateral Flow Strategy for a Fast Biosensing with an Improved Selectivity and Binding Affinity. Sensors.

[19] Zhao M., Wang X., Nolte D. (2010). Mass-transport limitations in spot-based microarrays. Biomed. Opt. Express.

[20] Yang J., Wang X., Sun Y., Chen B., Hu F., Guo C., Yang T. (2022). Recent Advances in Colorimetric Sensors Based on Gold Nanoparticles for Pathogen Detection. Biosensors.

[21] Aldewachi H., Chalati T., Woodroofe M.N., Bricklebank N., Sharrack B., Gardiner P. (2017). Gold nanoparticle-based colorimetric biosensors. Nanoscale.

[22] Yang X., Huang R., Xiong L., Chen F., Sun W., Yu L. (2023). A Colorimetric Aptasensor for Ochratoxin A Detection Based on Tetramethylrhodamine Charge Effect-Assisted Silver Enhancement. Biosensors.

[23] Pham X.H., Hahm E., Kim T.H., Kim H.M., Lee S.H., Lee Y.S., Jeong D.H., Jun B.H. (2018). Enzyme-catalyzed Ag Growth on Au Nanoparticle-assembled Structure for Highly Sensitive Colorimetric Immunoassay. Sci. Rep..

[24] Shah M.M., Ren W., Irudayaraj J., Sajini A.A., Ali M.I., Ahmad B. (2021). Colorimetric Detection of Organophosphate Pesticides Based on Acetylcholinesterase and Cysteamine Capped Gold Nanoparticles as Nanozyme. Sensors.

[25] Kubitschek H.E. (1990). Cell volume increase in *Escherichia coli* after shifts to richer media. J. Bacteriol..

[26] Park M. (2020). Surface Display Technology for Biosensor Applications: A Review. Sensors.

[27] Zhang B., Gao X., Zhou Y., You S., Qi W., Wang M. (2025). Surface Display Technologies for Whole-Cell Biocatalysts: Advances in Optimization Strategies, Food Applications, and Future Perspectives. Foods.

[28] Vazquez-Arias A., Perez-Juste J., Pastoriza-Santos I., Bodelon G. (2021). Prospects and applications of synergistic noble metal nanoparticle-bacterial hybrid systems. Nanoscale.

[29] Chen S., Chen X., Su H., Guo M., Liu H. (2023). Advances in Synthetic-Biology-Based Whole-Cell Biosensors: Principles, Genetic Modules, and Applications in Food Safety. Int. J. Mol. Sci..

[30] Soler M., Lechuga L.M. (2022). Biochemistry strategies for label-free optical sensor biofunctionalization: Advances towards real applicability. Anal. Bioanal. Chem..

[31] Zhang Y., Wu Y., Zhao X., Ye Q., Cao L., Liu M., Gao B., Niu Q., Chen N., Duan Z. (2025). Progress and challenges of functionalized bacterial encapsulation: A novel biotechnology for next-generation biotherapeutics. Acta Pharm. Sin. B.

[32] Gallus S., Mittmann E., Rabe K.S. (2022). A Modular System for the Rapid Comparison of Different Membrane Anchors for Surface Display on *Escherichia coli*. ChemBioChem.

[33] Weber P.C., Ohlendorf D.H., Wendoloski J.J., Salemme F.R. (1989). Structural origins of high-affinity biotin binding to streptavidin. Science.

[34] Livnah O., Bayer E.A., Wilchek M., Sussman J.L. (1993). Three-dimensional structures of avidin and the avidin-biotin complex. Proc. Natl. Acad. Sci. USA.

[35] Brambilla D., Sola L., Damin F., Mussida A., Chiari M. (2023). Immobilization of biotinylated antibodies through streptavidin binding aptamer. Talanta.

[36] Heinisch T., Schwizer F., Garabedian B., Csibra E., Jeschek M., Vallapurackal J., Pinheiro V.B., Marliere P., Panke S., Ward T.R. (2018). E. coli surface display of streptavidin for directed evolution of an allylic deallylase. Chem. Sci..

[37] Lin W.-Z., Wang J.-P., Ma I.-C., Hsieh P.-C., Hung Y.-J., Hung C.-M., Hou S.-Y. (2023). Highly sensitive β-galactosidase detection using streptavidin-display *E. coli* and lateral flow immunoassay. Sens. Actuators A Phys..

[38] Mateos H., Mallardi A., Serrano-Pertierra E., Blanco-López M.C., Izzi M., Cioffi N., Palazzo G. (2023). Unusual gold nanoparticle-antibody interactions. JCIS Open.

[39] Guerrini L., Alvarez-Puebla R.A., Pazos-Perez N. (2018). Surface Modifications of Nanoparticles for Stability in Biological Fluids. Materials.

[40] Xu X., Zhang M., Li Z., Ye D., Gou L., Zou Q., Zhu L. (2023). Highly efficient light-induced self-assembly of gold nanoparticles promoted by photoexcitation-induced aggregatable ligands. Chem. Commun..

[41] Sendroiu I.E., Mertens S.F., Schiffrin D.J. (2006). Plasmon interactions between gold nanoparticles in aqueous solution with controlled spatial separation. Phys. Chem. Chem. Phys..

[42] Zhang L., Tan L., Liu M., Chen Y., Yang Y., Zhang Y., Zhao G. (2024). Quantitative measurement of cell-surface displayed proteins based on split-GFP assembly. Microb. Cell Fact..

[43] Park M., Jose J., Thommes S., Kim J.I., Kang M.J., Pyun J.C. (2011). Autodisplay of streptavidin. Enzym. Microb. Technol..

[44] Cho I.H., Paek E.H., Lee H., Kang J.Y., Kim T.S., Paek S.H. (2007). Site-directed biotinylation of antibodies for controlled immobilization on solid surfaces. Anal. Biochem..

[45] Gao S., Guisan J.M., Rocha-Martin J. (2022). Oriented immobilization of antibodies onto sensing platforms—A critical review. Anal. Chim. Acta.

[46] De Michele C., De Los Rios P., Foffi G., Piazza F. (2016). Simulation and Theory of Antibody Binding to Crowded Antigen-Covered Surfaces. PLoS Comput. Biol..

[47] Hadzhieva M., Pashov A.D., Kaveri S., Lacroix-Desmazes S., Mouquet H., Dimitrov J.D. (2017). Impact of Antigen Density on the Binding Mechanism of IgG Antibodies. Sci. Rep..

[48] Blackburn S.A., Shepherd M., Robinson G.K. (2021). Reciprocal Packaging of the Main Structural Proteins of Type 1 Fimbriae and Flagella in the Outer Membrane Vesicles of “Wild Type” *Escherichia coli* Strains. Front. Microbiol..

[49] Wang J., Ma W., Wang X. (2021). Insights into the structure of *Escherichia coli* outer membrane as the target for engineering microbial cell factories. Microb. Cell Fact..

[50] Jung J., Sung J.S., Kim T.-H., Kang M.-J., Jose J., Shin H.-J., Pyun J.-C. (2024). One-Step Immunoassay for the Detection of SARS-CoV-2 Nucleocapsid Protein Using Screened Fv-Antibodies. BioChip J..

[51] Smith A.M., Borkovec M., Trefalt G. (2020). Forces between solid surfaces in aqueous electrolyte solutions. Adv. Colloid. Interface Sci..

[52] Bansal S.A., Kumar V., Karimi J., Singh A.P., Kumar S. (2020). Role of gold nanoparticles in advanced biomedical applications. Nanoscale Adv..

