# Supplementary material for: Streptavidin-Displaying E. coli Scaffolds Enable Target-Mediated Gold Nanoparticle Assembly for Colorimetric Biosensing
Source: Sensors (Basel). 2026 Sep 21;26(18):5965. doi: 10.3390/s26185965 (PMC13611320; doi:10.3390/s26185965)
Supplement: Supplementary file 1 [file sensors-26-05965-s001.zip › sensors-4555223-supplementary.pdf]

Supplementary Information

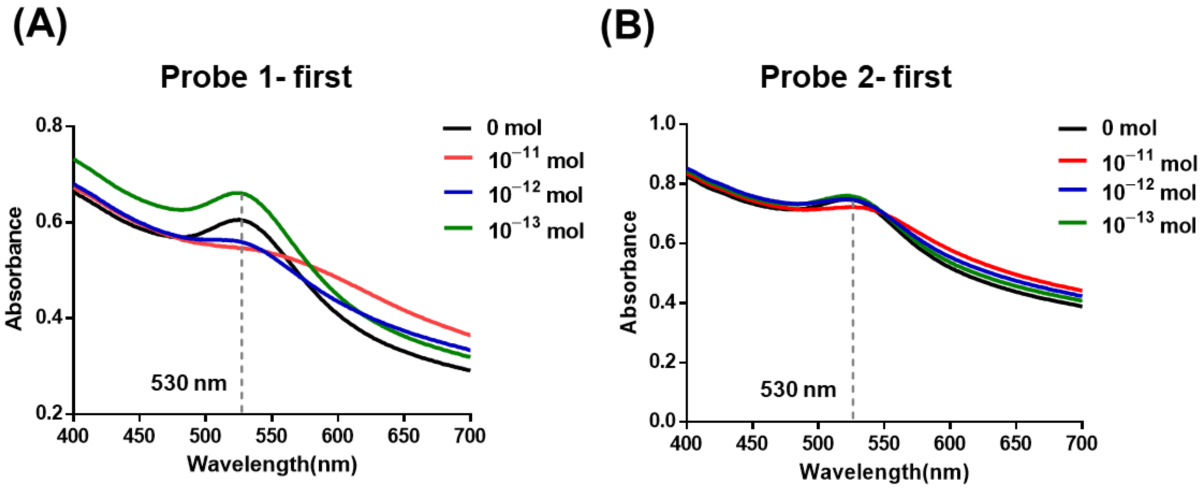

Supplementary Figure S1. UV-Vis absorbance spectra of the sensing system at different amounts of recombinant SARS-CoV-2 N protein with different probe-addition sequences. AuNP-Ab and *E. coli*-SA/Bio-Ab probes are denoted as Probe 1 and Probe 2, respectively. Spectra were obtained with 0, 10^{-13}, 10^{-12}, and 10^{-11} mol of recombinant SARS-CoV-2 N protein using (A) the Probe 1-first sequence and (B) the Probe 2-first sequence. The dashed lines indicate 530 nm.
